# Executive Functions and Performance Variability Measured by Event-Related Potentials to Understand the Neural Bases of Perceptual Decision-Making

**DOI:** 10.3389/fnhum.2017.00556

**Published:** 2017-11-15

**Authors:** Rinaldo L. Perri, Francesco Di Russo

**Affiliations:** ^1^Department Unicusano, University Niccolò Cusano, Rome, Italy; ^2^Department of Movement, Human and Health Sciences, Foro Italico University of Rome, Rome, Italy; ^3^IRCCS Santa Lucia Foundation, Rome, Italy

**Keywords:** event-related potentials (ERP), decision-making, executive functions, insula, response variability, electroencephalography, frontal lobe

## Deciding between different choices: neurocognitive factors of decision-making and response variability

Perceptual decision-making tasks usually require subjects to recognize stimulus categories and select between different response alternatives. For example, in Go/No-go tasks, one has to respond to target stimuli and withhold responding to non-target stimuli. Accomplishing even just a single trial of such a task needs a complex sequence of functions (most of them executive) consisting, for example, in motor readiness, sustained attention, sensory processing, inhibitory control, conflict monitoring, stimulus-response mapping, context updating and, if any, error detection and awareness. In this context, the motor response reflects the behavioral outcome of the fast and proper interaction of the above-mentioned processes, and the response consistency (or variability) is often adopted as index of executive functioning.

Nowadays, one challenge of the cognitive neuroscience is to understand how executive functions allow to make decisions. In fact, understanding decisional processes, and reasons of decision failure, would be helpful to clarify the executive dysfunctions of clinical conditions such as obsessive compulsive disorders, impulsivity, and addictions (typically intended as a failure of inhibition; Chamberlain et al., 2005; Crews and Boettiger, 2009; Álvarez-Moya et al., 2011), as well as success in real-life tasks (e.g., car driving; Bunce et al., 2012) and goal-directed behaviors (e.g., complying with diet schedules; Jahanshahi et al., 2015). In this context, the response variability reflects a behavioral index of efficiency of frontal cognitive control (Bellgrove et al., 2004), and this association was suggested since the first half of the twentieth century, when Head (1926, p. 145) reported that “*an inconsistent response is one of the most striking consequences of lesions to the cerebral cortex.”* More recently, consistent literature indicated response variability as an indirect index of top down control (Tamm et al., 2012), executive functioning (Swick et al., 2013), neurological (Segalowitz et al., 1997; Hultsch et al., 2000), and psychiatric conditions (Barkley et al., 1992; Vinogradov et al., 1998; Leth-Steensen et al., 2000), and frontal lobes integrity (Bunce et al., 2007; Walhovd and Fjell, 2007; Lövdén et al., 2013). The frontal cortex is in fact considered as the main region supporting the executive functions and behavioral variability (Stuss et al., 2003), as revealed by the poor response consistency and accuracy of frontal patients performing a decision-making task (Arnot, 1952; Stuss et al., 1999, 2003; Picton et al., 2007).

Even though it is evident the relationship between executive functioning and performance variability at group level (e.g., in the comparison between high- and low-level athletes in sport; Vestberg et al., 2012), it is less known the mediating role of response variability at intra-individual level. Also, it is still not clear the mediating role of PFC activity in the intra-individual variability because of contrasting results of neuroimaging studies: in fact, two studies reported a greater dorsolateral PFC (DLPFC) activation associated with high intra-individual variability (Bellgrove et al., 2004; Simmonds et al., 2007), while Weissman et al. (2006) reported reduced pre-stimulus activity of the right DLPFC in the less consistent trials. In other words, depending on the main findings, neuroimaging literature interpreted the high individual variability as the consequence of the greater need of top-down executive control (enhanced PFC activation), or in terms of lapses in attention (reduced PFC activation).

## ERPs and executive functions: state of the art and main limitations

Identification of neurophysiological correlates of executive functioning requires to investigate different cognitive abilities, which in part depend on the experimental paradigm: for example, in Stroop or sustained attention tasks (Demeter and Woldorff, 2016), voluntary selective attention would be more stressed than Go/No-go tasks in which accumulation of sensory evidence would be determinant, or oddball tasks where decision making in effected by expectancy, or stop-signal tasks in which the so-called “reactive inhibition” is often required (for a review see Jahanshahi et al., 2015). It is also relevant to note that decisional processes work in a narrow temporal window, such as the time needed to perform a single trial in a speeded decision-making task. This constraint requires the researchers to adopt a technique with adequate temporal resolution to carry out their own investigations: this means that neuroimaging studies may not be the most suitable to investigate the fast temporal succession of the decisional processes. Moreover, as also suggested by Bogacz et al. (2010), the duration of the decision processes can affect the amplitude of the BOLD signal, therefore functional magnetic resonance (fMRI) findings should be interpreted with caution when studying the decision-making. In other terms, it could be possible that the long time needed to make a decision (leading to large response variability) explains the greater PFC activation reported by some studies (Bellgrove et al., 2004; Simmonds et al., 2007). At the opposite, even if less informative on the anatomical source of the observed activities, the electroencephalography (EEG), and especially the event-related potentials (ERPs) technique, is particularly appropriate to catch the fast succession of the decisional brain's events. However, most of the ERP literature in this field focused on post-response activities like the central-parietal P3 (Segalowitz et al., 1997; Saville et al., 2011), and the error-negativity (Ne) and error positivity (Pe) in case of error commission (Falkenstein et al., 1991, 1995, 1996). Similarly, when focused on the pre-movement activities, electrophysiological studies mainly observed the frontal-medial modulation of the N2 component (Bokura et al., 2001; van Boxtel et al., 2001; Nieuwenhuis et al., 2003), whose functional role is still debated (Perri et al., 2015b; Di Russo et al., 2017). In other terms, the main limitation of ERP literature was the lack of a solid background on the contribution of the executive functions of the frontal cortex in the decisional processes. In fact, except for the motor preparation activities of the frontal areas, as reflected by the Bereitschaftspotential (BP; e.g., Shibasaki and Hallett, 2006) and the lateralized readiness potential (LRP; e.g., Rinkenauer et al., 2004), only the very recent literature started to report the ERP correlates of the PFC in the executive functioning and variability (for a review see Di Russo et al., 2017).

## Looking into the frontal lobes: emerging evidence on the ERP correlates of executive functions

When performing a decision-making task, the contribution of the PFC executive functions is manifested mainly through cognitive processes like top-down attentional control, maintenance of information in the short-term memory, ability to ignore distractors and focus on relevant features, and inhibition of the wrong schema and selection of the appropriate one. Since even one of these processes would be able to affect the inter- and intra-individual variability of the performance, they should be dissociated and investigated separately.

**Figure 1:**
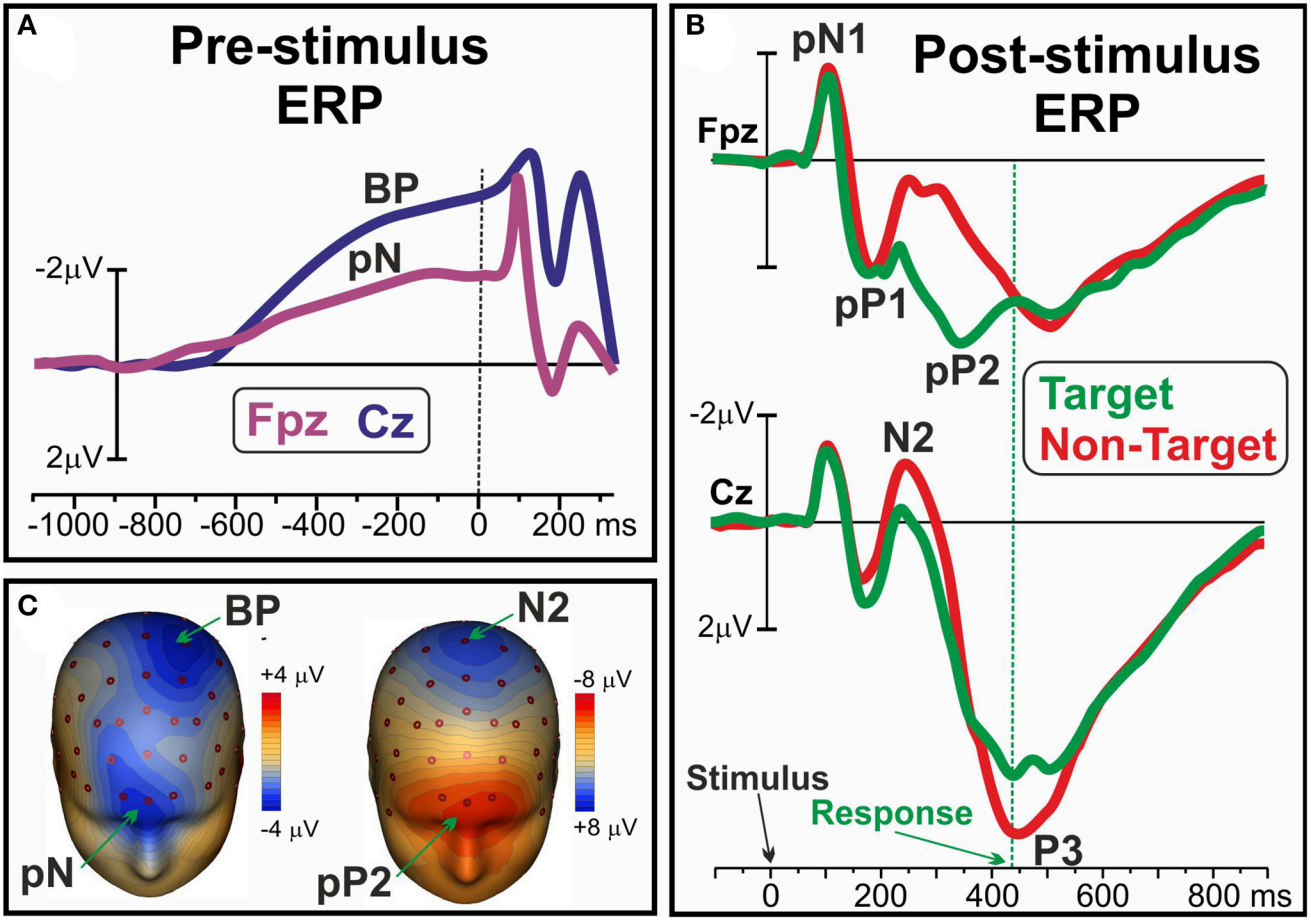
**(A)** Pre-stimulus ERP waveforms of the pN and BP components over medial prefrontal (Fpz) and frontal sites (Cz), respectively. **(B)** Post-stimulus pN1, pP1 and pP2 ERP components over Fpz; N2 and P3 components at Cz. **(C**) Scalp topography of the pN and BP (left), and of the pP2 and N2 (right). Data derived from Di Russo et al. (2016).

Recent ERP studies described different pre-movement activities within the frontal cortex emerging both before and after the stimulus appearance in decisional tasks: as reviewed by Di Russo et al. (2017), there is a growing body of evidence defining the mediating role of these components in the variability of executive functions. It was shown that before the appearance of a stimulus, that is the preparation stage, at frontopolar sites is possible to detect the so called prefrontal negativity (pN) component, as shown in Figure 1A and in the left side of Figure 1C, together with the more posterior BP (e.g., Di Russo et al., 2013). The bilaterally-distributed pN was described as the electrophysiological correlate of the inferior frontal gyrus (iFg) activity (Di Russo et al., 2016; Sulpizio et al., 2017), especially involved in the proactive inhibitory (Perri et al., 2016; Bianco et al., 2017a,b,c) and top-down attentional control (Perri et al., 2014a, 2015a, 2017). Another ERP component is the dorsolateral pN (DLpN), that on the right hemisphere was associated to modulation of baseline levels in the accuracy system (i.e., the larger the right DLpN, the poorer the accuracy performance; Perri et al., 2014b; Lucci et al., 2016). The mediating role of the pN component in intra-individual variability emerged through studies that showed how enhancement of this activity predisposes subjects to effective inhibitory control (Perri et al., 2016) and, at the opposite, that its reduction predicts poor attentional control leading to response omission (Perri et al., 2017). A prefrontal activity compatible with the pN was described by West and Alain (2000) adopting a Stroop task: findings are consistent in revealing that momentary lapses of attention are associated with a pre-stimulus change in the ERP activity over the frontal regions. Moreover, it was shown that also inter-individual variability of performance was associated with the individual level of pN activity: in other words, the more consistent performers are marked by larger pN activity than the less consistent ones (Perri et al., 2015a). If the pN component can be described as a sort of readiness activity (a cognitive disposition in performing the task), there are also executive functions that work in the information processing stage, that is after the stimulus onset. In this regard, the ERP literature identified a complex of three components that were labeled as prefrontal N1 (pN1), prefrontal P1 (pP1), and prefrontal P2 (pP2): they were respectively associated with the sensory-awareness, the sensory-motor integration and the stimulus-response mapping process (for a review see Di Russo et al., 2017). The main generator of the prefrontal ERPs was localized in the bilateral anterior insula (Di Russo et al., 2016; Sulpizio et al., 2017), and these components are typically detected 80–400 ms after the stimulus appearance; however, while the pN1 and pP1 components reflect top-down perceptual processing of any stimulus to be processed (even in a passive-perception task), the later pP2 can be detected only in presence of decisional requests, that is the need to classify the information by matching it with the relative response, or stimulus-response mapping. Figure 1B shows the ERP waveforms of the pN1, pP1 and Pp2 together with the well-known N2 and P3. Figure 1C (right) shows the pP2 scalp topography concomitant to the N2. It is noteworthy that the pP2 component was also labeled as Go-P2 (Gajewski and Falkenstein, 2013), anterior P2 (P2a; Potts et al., 1996), frontal selection positivity (FSP; Kenemans et al., 1993), and frontal P3 (P3f; Makeig et al., 1999), and in all cases larger amplitudes were reported for target than non-target trials, regardless of task and response modality (finger movement, speech, silent count). Further, modulation of this component were repeatedly associated with groups difference in decisional speed (Perri et al., 2014b; Bianco et al., 2017c), such as with accuracy variability at both inter-individual (Di Russo et al., 2013; Perri et al., 2014b), and intra-individual level (Perri et al., 2015b, 2017).

Concluding, there is a growing literature revealing the utility of the ERPs in the study of the executive functions of the prefrontal cortex. In fact, even if with a less spatial resolution of neuroimaging scans, recent ERP literature has proven to be able in overcoming some concerns such as the source localization and the presence of artifacts in the anterior sites, that in the past may have limited the EEG investigation of the frontal executive functioning. At the opposite, the background reviewed here on the functional roles and generators of prefrontal ERPs suggest the latter as a promising tool to foster a new-way approach in the neurocognitive study of the executive functions. In fact, the extensive review of Di Russo et al. (2017) revealed the potential role of the prefrontal ERPs in identifying the cognitive factors mediating the variability of performance within subjects and between groups. Also, since it was shown that prefrontal ERPs are affected by neurological disease and susceptible of modifications as effect of rehabilitation (Di Russo et al., 2013) and sport training (Bianco et al., 2017b,c), future studies may clarify which cognitive factors could operate on them. Similarly, investigation of the prefrontal ERPs in clinical populations would be useful to shed new light on the strength relationship between prefrontal lesions and executive functions (for a review see Alvarez and Emory, 2006).

## Author contributions

RP: conception and writing of the work; FD: contribution to writing and conception of the work, critical revision of the text; RP and FD: approved the final version of the manuscript.

### Conflict of interest statement

The authors declare that the research was conducted in the absence of any commercial or financial relationships that could be construed as a potential conflict of interest.

## References

[B1] Alvarez J. A. Emory E. (2006). Executive function and the frontal lobes: a meta-analytic review. Neuropsychol. Rev. 16, 17–42. 10.1007/s11065-006-9002-x 16794878

[B2] Arnot R. (1952). A theory of frontal lobe function. Arch. Neurol. Psychiatry 67:487. 10.1001/archneurpsyc.1952.02320160071008 14914227

[B3] Barkley R. A. Grodzinsky G. DuPaul G. J. (1992). Frontal lobe functions in attention deficit disorder with and without hyperactivity: a review and research report. J. Abnorm. Child Psychol. 20, 163–188. 10.1007/BF00916547 1593025

[B4] Bellgrove M. A. Hester R. Garavan H. (2004). The functional neuroanatomical correlates of response variability: evidence from a response inhibition task. Neuropsychologia 42, 1910–1916. 10.1016/j.neuropsychologia.2004.05.007 15381021

[B5] Bianco V. Berchicci M. Perri R. L. Quinzi F. Di Russo F. (2017a). Exercise-related cognitive effects on sensory-motor control in athletes and drummers compared to non-athletes and other musicians. Neuroscience 360, 39–47. 10.1016/j.neuroscience.2017.07.059 28764939

[B6] Bianco V. Berchicci M. Perri R. L. Spinelli D. Di Russo F. (2017b). The proactive self-control of actions: time-course of underlying brain activities. Neuroimage 156, 388–393 10.1016/j.neuroimage.2017.05.043 28533119

[B7] Bianco V. Di Russo F. Perri R. L. Berchicci M. (2017c). Different proactive and reactive action control in fencers' and boxers' brain. Neuroscience 343, 260–268. 10.1016/j.neuroscience.2016.12.006 28003155

[B8] Bogacz R. Wagenmakers E. J. Forstmann B. U. Nieuwenhuis S. (2010). The neural basis of the speed-accuracy tradeoff. Trends Neurosci. 33, 10–16. 10.1016/j.tins.2009.09.002 19819033

[B9] Bokura H. Yamaguchi S. Kobayashi S. (2001). Electrophysiological correlates for response inhibition in a Go/NoGo task. Clin. Neurophysiol. 112, 2224–2232. 10.1016/S1388-2457(01)00691-5 11738192

[B10] Bunce D. Anstey K. J. Christensen H. Dear K. Wen W. Sachdev P. (2007). White matter hyperintensities and within-person variability in community-dwelling adults aged 60–64 years. Neuropsychologia 45, 2009–2015. 10.1016/j.neuropsychologia.2007.02.006 17382358

[B11] Bunce D. Young M. S. Blane A. Khugputh P. (2012). Age and inconsistency in driving performance. Accid. Anal. Prev. 49, 293–299. 10.1016/j.aap.2012.01.001 23036409

[B12] Chamberlain S. R. Blackwell A. D. Fineberg N. A. Robbins T. W. Sahakian B. J. (2005). The neuropsychology of obsessive compulsive disorder: the importance of failures in cognitive and behavioural inhibition as candidate endophenotypic markers. Neurosci. Biobehav. Rev. 29, 399–419. 10.1016/j.neubiorev.2004.11.006 15820546

[B13] Crews F. T. Boettiger C. A. (2009). Impulsivity, frontal lobes and risk for addiction. Pharmacol. Biochem. Behav. 93, 237–247. 10.1016/j.pbb.2009.04.018 19410598 PMC2730661

[B14] Demeter E. Woldorff M. G. (2016). Transient distraction and attentional control during a sustained selective attention task. J. Cogn. Neurosci. 28, 935–947. 10.1162/jocn_a_00949 26967946 PMC4887321

[B15] Di Russo F. Berchicci M. Bozzacchi C. Perri R. L. Pitzalis S. Spinelli D. (2017). Beyond the “Bereitschaftspotential:” action preparation behind cognitive functions. Neurosci. Biobehav. Rev. 78, 57–81. 10.1016/j.neubiorev.2017.04.019 28445742

[B16] Di Russo F. Berchicci M. Perri R. L. Ripani F. R. Ripani M. (2013). A passive exoskeleton can push your life up: application on multiple sclerosis patients. PLoS ONE 8:e77348 10.1371/journal.pone.0077348 24204814 PMC3808392

[B17] Di Russo F. Lucci G. Sulpizio V. Berchicci M. Spinelli D. Pitzalis S.. (2016). Spatiotemporal brain mapping during preparation, perception, and action. Neuroimage 126, 1–14. 10.1016/j.neuroimage.2015.11.036 26608247

[B18] Falkenstein M. Hohnsbein J. Hoormann J. (1995). Event-related potential correlates of errors in reaction tasks. Electroencephal. Clin. Neurophysiol. Suppl. 44, 287–296. 7649035

[B19] Falkenstein M. Hohnsbein J. Hoormann J. (1996). Differential processing of motor errors, in Recent Advance Event-Related Brain Potential Research, eds Ogura C. Koga Y. Shimokochi M. (Amsterdam: Elsevier Science), 579–585.

[B20] Falkenstein M. Hohnsbein J. Hoormann J. Blanke L. (1991). Effects of crossmodal divided attention on late ERP components. II. error processing in choice reaction tasks. Electroencephal. Clin. Neurophysiol. 78, 447–455. 10.1016/0013-4694(91)90062-9 1712280

[B21] Gajewski P. D. Falkenstein M. (2013). Effects of task complexity on ERP components in Go/No-go tasks. Int. J. Psychophysiol. 87, 273–278. 10.1016/j.ijpsycho.2012.08.007 22906814

[B22] Head H. (1926). Aphasia and Kindred Disorders of Speech. Cambridge: Cambridge University Press.

[B23] Hultsch D. F. MacDonald S. W. Hunter M. A. Levy-Bencheton J. Strauss E. (2000). Intraindividual variability in cognitive performance in older adults: comparison of adults with mild dementia, adults with arthritis, and healthy adults. Neuropsychology 14:588. 10.1037/0894-4105.14.4.588 11055261

[B24] Jahanshahi M. Obeso I. Rothwell J. C. Obeso J. A. (2015). A fronto-striato-subthalamic-pallidal network for goal-directed and habitual inhibition. Nat. Rev. Neurosci. 16:719. 10.1038/nrn4038 26530468

[B25] Kenemans J. L. Kok A. Smulders F. T. Y. (1993). Event-related potentials to conjunctions of spatial frequency and orientation as a function of stimulus parameters and response requirements. Electroencephalogr. Clin. Neurophysiol. 88, 51–63. 10.1016/0168-5597(93)90028-N 7681391

[B26] Leth-Steensen C. King Elbaz Z. Douglas V. I. (2000). Mean response times, variability, and skew in the responding of ADHD children: a response time distributional approach. Acta Psychol. 104, 167–190. 10.1016/S0001-6918(00)00019-6 10900704

[B27] Lövdén M. Schmiedek F. Kennedy K. M. Rodrigue K. M. Lindenberger U. Raz N. (2013). Does variability in cognitive performance correlate with frontal brain volume? Neuroimage 64, 209–215. 10.1016/j.neuroimage.2012.09.039 23000256 PMC3508369

[B28] Lucci G. Berchicci M. Perri R. L. Spinelli D. Di Russo F. (2016). Effect of target probability on pre-stimulus brain activity. Neuroscience 322, 121–128. 10.1016/j.neuroscience.2016.02.029 26912279

[B29] Makeig S. Westerfield M. Jung T. P. Covington J. Townsend J. Sejnowski T. J.. (1999). Functionally independent components of the late positive event-related potential during visual spatial attention. J. Neurosci. 19, 2665–2680. 10087080 10.1523/JNEUROSCI.19-07-02665.1999 PMC6786079

[B30] Álvarez-Moya E. M. Ochoa C. Jiménez-Murcia S. Aymamí M. N. Gómez-Pea M. Fernández-Aranda F.. (2011). Effect of executive functioning, decision-making and self-reported impulsivity on the treatment outcome of pathologic gambling. J. Psychiatry Neurosci. 36:165. 10.1503/jpn.090095 21138656 PMC3080512

[B31] Nieuwenhuis S. Yeung N. Van Den Wildenberg W. Ridderinkhof K. R. (2003). Electrophysiological correlates of anterior cingulate function in a Go/No-go task: effects of response conflict and trial type frequency. Cogn. Affect. Behav. Neurosci. 3, 17–26. 10.3758/CABN.3.1.17 12822595

[B32] Perri R. L. Berchicci M. Lucci G. Cimmino R. L. Bello A. Di Russo F. (2014a). Getting ready for an emotion: specific premotor brain activities for self-administered emotional pictures. Front. Behav. Neurosci. 8:197. 10.3389/fnbeh.2014.00197 24904344 PMC4035832

[B33] Perri R. L. Berchicci M. Lucci G. Spinelli D. Di Russo F. (2015a). The premotor role of the prefrontal cortex in response consistency. Neuropsychology 29:767. 10.1037/neu0000168 25528609

[B34] Perri R. L. Berchicci M. Lucci G. Spinelli D. Di Russo F. (2015b). Why do we make mistakes? neurocognitive processes during the preparation-perception-action cycle and error-detection. Neuroimage 113, 320–328. 10.1016/j.neuroimage.2015.03.040 25812715

[B35] Perri R. L. Berchicci M. Lucci G. Spinelli D. Di Russo F. (2016). How the brain prevents a second error in a perceptual decision making task. Sci. Rep. 6:32058. 10.1038/srep32058 27534593 PMC4989192

[B36] Perri R. L. Berchicci M. Spinelli D. Di Russo F. (2014b). Individual differences in response speed and accuracy are associated to specific brain activities of two interacting systems. Front. Behav. Neurosci. 8:251. 10.3389/fnbeh.2014.00251 25100961 PMC4106455

[B37] Perri R. L. Spinelli D. Di Russo F. (2017). Missing the target: the neural processing underlying the omission error. Brain Topogr. 30, 352–363. 10.1007/s10548-017-0545-3 28108852

[B38] Picton T. W. Stuss D. T. Alexander M. P. Shallice T. Binns M. A. Gillingham S. (2007). Effects of focal frontal lesions on response inhibition. Cereb. Cortex 17, 826–838. 10.1093/cercor/bhk031 16699079

[B39] Potts G. F. Liotti M. Tucker D. M. Posner M. I. (1996). Frontal and inferior temporal cortical activity in visual target detection: evidence from high spatially sampled event-related potentials. Brain Topogr. 9, 3–14. 10.1007/BF01191637

[B40] Rinkenauer G. Osman A. Ulrich R. Müller-Gethmann H. Mattes S. (2004). On the locus of speed-accuracy trade-off in reaction time: inferences from the lateralized readiness potential. J. Exp. Psychol. Gen. 133:261. 10.1037/0096-3445.133.2.261 15149253

[B41] Saville C. W. Dean R. O. Daley D. Intriligator J. Boehm S. Feige B.. (2011). Electrocortical correlates of intra-subject variability in reaction times: average and single-trial analyses. Biol. Psychol. 87, 74–83. 10.1016/j.biopsycho.2011.02.005 21335053

[B42] Segalowitz S. J. Dywan J. Unsal A. (1997). Attentional factors in response time variability after traumatic brain injury: an ERP study. J. Int. Neuropsychol. Soc. 3, 95–107. 9126851

[B43] Shibasaki H. Hallett M. (2006). What is the Bereitschaftspotential?. Clin. Neurophysiol. 117, 2341–2356. 10.1016/j.clinph.2006.04.025 16876476

[B44] Simmonds D. J. Fotedar S. G. Suskauer S. J. Pekar J. J. Denckla M. B. Mostofsky S. H. (2007). Functional brain correlates of response time variability in children. Neuropsychologia 45, 2147–2157. 10.1016/j.neuropsychologia.2007.01.013 17350054

[B45] Stuss D. T. Murphy K. J. Binns M. A. (1999). The frontal lobes and performance variability: evidence from reaction time. J. Int. Neuropsychol. Soc. 5:123.

[B46] Stuss D. T. Murphy K. J. Binns M. A. Alexander M. P. (2003). Staying on the job: the frontal lobes control individual performance variability. Brain 126, 2363–2380. 10.1093/brain/awg237 12876148

[B47] Sulpizio V. Lucci G. Berchicci M. Galati G. Pitzalis S. Di Russo F. (2017). Hemispheric asymmetries in the transition from action preparation to execution. Neuroimage 148, 390–402. 10.1016/j.neuroimage.2017.01.009 28069542

[B48] Swick D. Honzel N. Larsen J. Ashley V. (2013). Increased response variability as a marker of executive dysfunction in veterans with post-traumatic stress disorder. Neuropsychologia 51, 3033–3040. 10.1016/j.neuropsychologia.2013.10.008 24157540 PMC4529278

[B49] Tamm L. Narad M. E. Antonini T. N. O'Brien K. M. Hawk L. W. Jr. Epstein J. N. (2012). Reaction time variability in ADHD: a review. Neurotherapeutics 9, 500–508. 10.1007/s13311-012-0138-5 22930417 PMC3441931

[B50] van Boxtel G. J. van der Molen M. W. Jennings J. R. Brunia C. H. (2001). A psychophysiological analysis of inhibitory motor control in the stop-signal paradigm. Biol. Psychol. 58, 229–262. 10.1016/S0301-0511(01)00117-X 11698116

[B51] Vestberg T. Gustafson R. Maurex L. Ingvar M. Petrovic P. (2012). Executive functions predict the success of top-soccer players. PLoS ONE 7:e34731. 10.1371/journal.pone.0034731 22496850 PMC3319604

[B52] Vinogradov S. Poole J. H. Willis-Shore J. Ober B. A. Shenaut G. K. (1998). Slower and more variable reaction times in schizophrenia: what do they signify? Schizophr. Res. 32, 183–190. 10.1016/S0920-9964(98)00043-7 9720123

[B53] Walhovd K. B. Fjell A. M. (2007). White matter volume predicts reaction time instability. Neuropsychologia 45, 2277–2284. 10.1016/j.neuropsychologia.2007.02.022 17428508

[B54] Weissman D. H. Roberts K. C. Visscher K. M. Woldorff M. G. (2006). The neural bases of momentary lapses in attention. Nat. Neurosci. 9, 971–978. 10.1038/nn1727 16767087

[B55] West R. Alain C. (2000). Evidence for the transient nature of a neural system supporting goal-directed action. Cereb. Cortex 10, 748–752. 10.1093/cercor/10.8.748 10920047

